# Impact Dynamics and Freezing Performance of Porcine Bile Droplets on Horizontal Cold Substrates: Towards Advanced and Sustainable Food Processing

**DOI:** 10.3390/foods14132173

**Published:** 2025-06-21

**Authors:** Xinkang Hu, Bo Zhang, Libang Chen, Zhenpeng Zhang, Huanhuan Zhang, Xintong Du, Xu Wang, Lulu Zhang, Tao Yang, Chundu Wu

**Affiliations:** 1 School of Agricultural Engineering, Jiangsu University, Zhenjiang 212013, China; huxk@stmail.ujs.edu.cn (X.H.); 2212216014@stmail.ujs.edu.cn (L.C.); 2212316063@stmail.ujs.edu.cn (Z.Z.); 2111916002@stmail.ujs.edu.cn (H.Z.); 2112316032@stmail.ujs.edu.cn (X.W.); zhangll@stmail.ujs.edu.cn (L.Z.); 2212316058@stmail.ujs.edu.cn (T.Y.); 2 Key Laboratory for Theory and Technology of Intelligent Agricultural Machinery and Equipment, Jiangsu University, Zhenjiang 212013, China; 3 Jiangsu Province and Education Ministry Co-Sponsored Synergistic Innovation Center of Modern Agricultural Equipment, Jiangsu University, Zhenjiang 212013, China; 4 School of the Environment and Safety Engineering, Jiangsu University, Zhenjiang 212013, China; 2112209003@stmail.ujs.edu.cn

**Keywords:** agro-byproducts processing, cryogenic technology, impact dynamics, freezing behaviour, multiphysical interactions

## Abstract

With the development of the agro-processing industry, the efficient cryogenic treatment and resource utilization of porcine bile—a high-value byproduct—has received increasing attention. This study investigates the dynamic behaviour and freezing characteristics of porcine bile droplets upon impact on cold substrates under varying conditions of surface temperature (−10 °C to −20 °C) and impact velocity (0.18–0.59 m/s). The effects of droplet size, dimensionless numbers (Weber, Reynolds, Bond, Ohnesorge, and Prandtl), and thermal gradients were systematically analyzed. A thermoelectric cooling substrate combined with high-speed imaging was used to quantitatively characterize the spreading ratio, retraction ratio, and freezing time of droplets. The results show that the maximum spreading ratio increases with higher impact velocity but decreases with lower substrate temperature. Lower substrate temperatures significantly shorten the freezing time, with a maximum reduction of up to 45%, particularly for smaller droplets. Droplets with high Weber numbers (*We* > 3) form flattened ice layers with preserved retraction patterns, while those with low Weber numbers (*We* < 1) generate smooth, hemispherical ice caps. For the first time, the thermophysical properties of porcine bile were incorporated into the framework of droplet impact dynamics on cryogenic surfaces. The findings reveal multiscale freezing mechanisms of biological fluids at low temperatures and provide a theoretical basis for optimizing processes such as freeze-drying and cryogenic sterilization in agro-product processing.

## 1. Introduction

With the continued growth of the global population and rising levels of consumption, the agro-byproducts processing industry is experiencing unprecedented development opportunities [1]. This sector not only meets the increasing demand for diversified, nutritious, and convenient food products [2], but also enhances the added value of agricultural byproducts [3], driving the transformation and upgrading of the agricultural economy [4]. Current development trends emphasize green and sustainable practices [5], technological innovation, and the customization of products to meet personalized needs [6,7].

Porcine bile, a major by-product of liquid agricultural processing [8], is widely utilized in its freeze-dried or spray freeze-dried form as bile powder. This product serves as a functional food additive—commonly incorporated into laxative health supplements for its moistening properties to support intestinal regulation. Additionally, it is used in traditional medicinal food applications, often combined with honey to formulate granules or oral solutions for constipation relief, or added to herbal teas and detox beverages as a heat-clearing component [9]. With an annual global processing volume exceeding 300,000 tons [10], its efficient treatment and resource recovery are essential for increasing product value and minimizing environmental impact [11]. Freezing technologies are widely recognized as effective methods for improving the added value and shelf life of agricultural products [12]. In processes such as vacuum freeze-drying [13], spray freeze-drying [14], and low-temperature concentration [15], the dynamic interaction between liquid agro-product droplets and cryogenic substrates critically influences product uniformity, crystallization quality, and energy efficiency [16].Due to its unique fluid properties, including high viscosity and low surface tension [17], porcine bile exhibits markedly different impact and freezing dynamics compared to conventional fluids such as water [18]. However, systematic investigations into these differences remain limited. A comprehensive understanding of the impact-freezing behaviour of porcine bile droplets is therefore crucial—not only for optimizing agro-byproducts processing techniques, but also for advancing the development of green and high-performance cryogenic technologies for biological fluids.

In recent years, research on the dynamics and freezing behaviour of droplets impacting cryogenic surfaces has primarily focused on pure water droplets, yielding significant advances. Despite being a common phenomenon, the impact of a single droplet on a surface with a temperature below its freezing point remains highly complex. This process involves a range of coupled physical phenomena, including droplet spreading dynamics and moving contact lines [19], surface wettability [20], heat transfer between the spreading or oscillating droplet and the cold substrate [21], and solid-phase nucleation during freezing [22]. These phenomena are now widely understood to arise from the interplay among inertial, viscous, and capillary forces, as well as heat transfer and phase change processes. This interplay governs the droplet’s impact dynamics and ultimately influences its freezing morphology and efficiency. Several theoretical models have been developed to describe these mechanisms. Xu et al. [23] and Yang et al. [24] established models for water droplet spreading, retraction, and freezing, highlighting the regulatory role of dimensionless parameters such as the Weber number (*We*) and Reynolds number (*Re*) in liquid film evolution. Teske et al. [25] further investigated the heat transfer behaviour of viscous droplets under cryogenic conditions. The importance of considering the collective interaction of droplets in the freezing model is emphasized to improve the prediction accuracy in applications such as cryogenic droplet impact analysis.

However, for complex fluids such as porcine bile—an approximately Newtonian fluid with heterogeneous composition—the impact dynamics and freezing characteristics upon contact with cold substrates remain insufficiently studied. While some efforts have explored the physical properties of such droplets and reported engineering applications, systematic analysis is still lacking. For example, Amrit et al. [26] examined viscous Jatropha biodiesel emulsions and found that increasing water content reduced the maximum spreading diameter due to enhanced viscous effects, with implications for fuel injection system optimization. Goede et al. [27] developed a splashing model to predict the behaviour of shear-thinning fluids like blood, now widely applied in forensic science. Lin et al. [28] investigated the impact dynamics of droplets with varying viscosities on solid surfaces of different wettabilities and proposed a universal scaling law for spreading time, with applications in precision deposition during 3D printing and agricultural spraying [29,30]. Despite these advances, few experimental studies have systematically addressed porcine bile in the context of food freezing or pharmaceutical spray drying. Existing literature often overlooks the synergistic effects of fluid physical properties (e.g., surface tension and viscosity) and substrate temperature, relying instead on single-variable analysis. This limits the ability to elucidate the multiscale physical interactions involved. Three major knowledge gaps hinder the development of precision cryogenic technologies for liquid agro-products such as porcine bile: (1) the quantitative impact of high viscosity and low surface tension on spreading, retraction, and freezing behaviours remains unclear; (2) the coupling mechanisms between substrate temperature and droplet impact velocity lack experimental validation; (3) the dynamic competition between heat conduction and latent heat release during phase change in biological fluids is poorly understood. These limitations significantly constrain the refinement and optimization of low-temperature processing technologies for complex liquid agro-byproducts.

This study aims to systematically investigate the dynamic behaviour and freezing characteristics of porcine bile droplets impacting cryogenic substrates under varying surface temperatures (−10 °C to −20 °C) and impact velocities (0.18–0.59 m/s). The focus is on elucidating how droplet size, dimensionless parameters (e.g., Weber, Reynolds, and Prandtl numbers), and temperature gradients influence the freezing morphology and duration. It is hypothesized that droplet spreading increases with higher impact velocity, while lower substrate temperatures suppress retraction behaviour by accelerating heat dissipation, thereby reducing the total freezing time.An experimental setup was developed, consisting of a thermoelectric cooling substrate, a high-speed imaging system, and a droplet generation module. Impact velocity was controlled by adjusting the droplet release height (10–30 mm), and image analysis techniques were used to quantify spreading coefficient, retraction ratio, and freezing time. Five key dimensionless numbers—Weber (*We*), Reynolds (*Re*), Bond (*Bo*), Ohnesorge (*Oh*), and Prandtl (*Pr*)—were introduced to establish the correlation between droplet dynamics and freezing performance. The multifactorial interactions among these variables were systematically analyzed. This work represents the first incorporation of the thermophysical properties of porcine bile into a droplet impact dynamics framework on cryogenic surfaces, enriching the theoretical understanding of biological fluid behaviour at low temperatures. The findings offer a foundation for optimizing freeze-drying and cryo-pasteurization processes for liquid agro-products such as porcine bile. Additionally, the study provides a novel perspective for heat transfer research in multiphase flows involving complex, near-Newtonian fluids, with significant implications for both engineering applications and scientific advancement.

## 2. Materials and Methods

### 2.1. Materials and Pretreatment

Fresh porcine bile samples were obtained from healthy adult three-way crossbred pigs at certified slaughterhouse facilities (Zhenjiang Runjiang Slaughter Co., Ltd., Zhenjiang, Jiangsu, China) under veterinary supervision. Bile was aseptically aspirated from the gallbladder within 30 min post-euthanasia using sterile syringes to minimize contamination. All procedures adhered to local regulations governing the use of animal-derived materials. The physical properties of porcine bile were characterized as follows: density was measured using a Coriolis mass flow density meter (Emerson Electric Co., St. Louis, MO, USA); dynamic viscosity was determined with a BROOKFIELD R/S rheometer (Harlow, Essex, UK);measurements were performed at a shear rate range of 100–1000 s^−1^ (covering typical impact conditions). surface tension was assessed using a CAM 101 automatic tensiometer (KSV, Espoo, Finland); and specific heat capacity was measured using the HC2000 series flow-type calorimeter (Xi’an Xiaqxi Electronics Technology Co., Ltd., Xi’an, Shaanxi, China). A summary of the physical properties of porcine bile is presented in Table 1.

### 2.2. Experimental Setup

**Figure 1:**
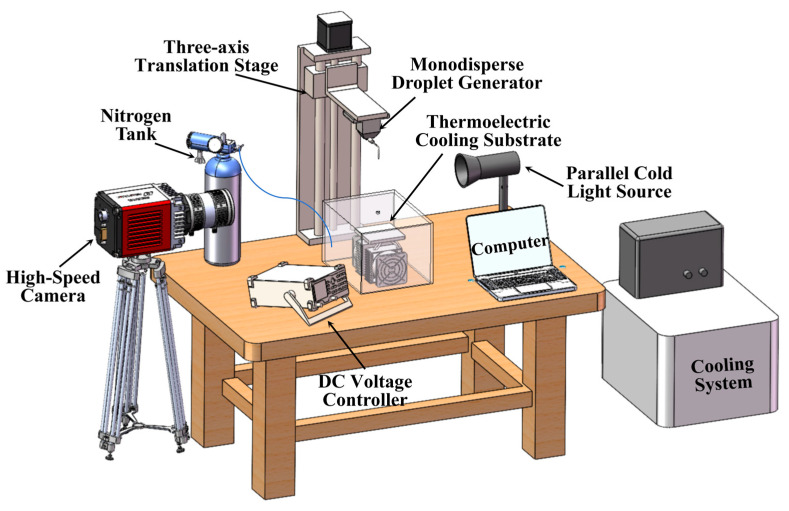
Experimental system diagram.

To ensure the validity and reliability of the experimental results, a custom-designed apparatus was developed to minimize foreseeable disturbances. As illustrated in Figure 1, the system consists of four primary modules: (1) a thermoelectric cooling substrate, (2) a droplet generation module, (3) an image acquisition and data processing module, and (4) a performance validation module.

The thermoelectric cooling substrate and its associated cooling system were designed to precisely regulate the surface temperature of the impact substrate. The assembly consists of an aluminum base plate, a thermoelectric cooler (TEC), a DC voltage controller (with a regulation precision of 0.1 V), and two air-cooled heat sinks. Both the aluminum plate and the TEC measure 100 mm × 100 mm in length and width. The aluminum substrate has a thickness of 2 mm, while the TEC unit is 4 mm thick and provides a maximum cooling power of 80 W. The cold side of the TEC is in direct contact with the aluminum substrate, whereas the hot side is coupled to the air-cooled heat sinks. To enhance thermal conductivity and reduce interfacial thermal resistance, a uniform layer of thermal grease was applied to both the hot and cold interfaces. The air-cooled heat sinks dissipate the heat generated by the TEC, ensuring the system operates under stable thermal conditions throughout the experiment.

The droplet generation module consists of a monodisperse droplet generator (Model MDG-100, TSI Inc., Shoreview, MN, USA) and a custom-built three-axis translation stage. This setup enables the controlled release of porcine biledroplets from variable heights to generate different impact velocities. Two characteristic porcine bile droplet sizes were used in this study: *D_Vs_* = 532 μm, and *D_Vs_* = 1028 μm. Monodisperse porcine bile droplets were produced by applying a square wave signal to the fluid ejection chamber of the generator. When the frequency of the signal matched the resonance frequency of the system, stable, uniformly sized droplets were generated. The working fluid was porcine bile, which exhibits distinct physicochemical properties compared to water, including a surface tension of 0.04471 N/m and a dynamic viscosity of 2.389 mPa·s. The droplet generator was mounted on a height-adjustable displacement platform, allowing precise control of the initial release height. This setup enabled the generation of droplets with tunable impact velocities upon contact with the cryogenic substrate.

The image acquisition and data processing module consists of a high-speed camera (SH6-116, Deep Vision Intelligent Technology Co., Ltd., Shenzhen, China), a cold light source, and the associated Fastphoto software v4.0 system. The high-speed camera was mounted on the side of the cryogenic substrate to capture the entire impact and freezing process of the porcine bile droplets from a lateral perspective. It operated at frame rates ranging from 1000 to 15,800 fps with a pixel size of 14.6 μm, allowing for precise image capture. The recorded image data were synchronized and stored on a computer, facilitating subsequent analysis and data processing. A parallel cold light source was used as the background lighting to enhance image brightness, ensuring high-quality visual documentation of the porcine bile droplet dynamics. The Fastphoto software system (Deep Vision Intelligent Technology Co., Ltd.) enabled efficient recording, playback, and data processing. For each data point, a minimum of 3 independent replicates were conducted using monodisperse porcine bile droplets with controlled diameters and release heights. During each run, the key parameters—such as maximum spreading diameter, oscillation time, and total freezing time—were extracted from high-speed image sequences using calibrated image analysis software. These standard deviations were used as vertical error bars to reflect the experimental variability due to factors such as droplet generation inconsistencies, substrate surface uniformity, or image resolution limits which provides a quantitative assessment of data reproducibility and reliability.

The performance testing module is designed to evaluate the horizontal stability and temperature retention of the cryogenic substrate. It consists of a leveling structure, a spirit level, and two surface-mounted temperature sensors. The leveling structure is used for fine adjustment of the substrate’s horizontal alignment. By rotating the nuts on the sandwich layer, the plane height can be finely tuned, while the spirit level ensures the substrate’s horizontal orientation. One temperature sensor is fixed on the cold surface of the substrate, and the other is installed inside the acrylic chamber to monitor the ambient temperature in real-time. The temperature sensors provide an accuracy of ±0.1 °C. Additionally, to prevent condensation or frost formation from ambient humidity on the cooling surface during the experiment, a 150 mm × 150 mm × 150 mm acrylic enclosure was designed. This enclosure is continuously purged with nitrogen gas before and throughout the experiment to maintain a dry environment.

### 2.3. Experimental Procedure and Conditions

All experiments were conducted at an ambient temperature of 20 ± 0.5 °C, with a relative humidity of 20 ± 1%(humidity control adopts MSSHIMEI MS-860D rotary wheel dehumidifier, (Shanghai, China)), and under standard atmospheric pressure. The experimental procedure began by leveling the cryogenic substrate. Nitrogen gas was introduced into the acrylic enclosure to maintain a dry environment. The DC voltage controller and its associated cooling system were then activated, and the substrate temperature was allowed to stabilize at the required experimental temperature. Once the desired temperature was achieved, the three-dimensional displacement stage was adjusted to the specified release height, ensuring that the droplets would fall at the center of the high-speed camera’s field of view. Next, temperature measurements were taken at the four corners of the droplet impact area using temperature sensors. The maximum temperature difference between these measurements was found to be less than 0.3 °C, indicating uniformity in the cold substrate temperature within the impact region. Subsequently, the high-speed camera and droplet generator were activated, and the droplet impact and freezing processes were recorded using the Fastphoto software system. After each experiment, compressed air was used to perform high-pressure purging and cleaning of the cold substrate, minimizing any potential damage to the surface.

The high-speed camera was set to a fixed frame rate of 2000 fps, with a resolution of 1028 × 1024 px and an exposure time of 100 μs. The field of view was fixed at a 10 mm × 10 mm area. To ensure real-time image and video data transmission, a 10-gigabit Ethernet connection was utilized, along with an adaptive 2.5 Gbps system. The data storage was supported by a 5 TB memory capacity to accommodate the extended recording durations required for the experiments.

The impact dynamics and freezing performance of porcine bile droplets on the cryogenic substrate are influenced by the material properties, physical parameters, and operational conditions of the porcine bile droplets. These include the surface tension (*σ*_l_), viscosity (*µ*_l_), density (*ρ*_l_), equivalent droplet diameter (*D*_0_), impact velocity (*v*_0_), diameter of the wetting region (*d_moment_*), specific heat capacity (*C*_l_), thermal conductivity (*k*_l_), and the surface temperature of the cold substrate (*T_s_*). The subscript “l” and “s” denote the liquid and solid phases, respectively. To describe the experimental conditions and capture the multi-scale physical effects, commonly used five-dimensional dimensionless numbers from fluid dynamics and heat transfer are employed.

The Weber number (*We*) represents the ratio of inertial forces to surface tension. When *We* ≫ 1, inertial forces dominate, and the droplet or fluid interface is prone to deformation or even rupture. Conversely, when *We* ≪ 1, surface tension dominates, leading to a stable fluid interface where droplets or bubbles are more likely to maintain a spherical shape.

(1) $$We=\frac{\rho_{l}v_{0}^{2}D_{0}}{\sigma_{l}}$$

The Reynolds number (*Re*) represents the ratio of inertial forces to viscous forces. For Reynolds numbers in the range 0 < *Re* ≤ 200, droplet motion is dominated by viscous forces, resulting in laminar flow with significant energy dissipation. Under these conditions, the spreading velocity is slow, and the retraction process is substantially limited. When *Re* > 200, inertial forces become more prominent, and the flow may transition toward a mixed or turbulent state. In this regime, the droplet’s kinetic energy plays a more pronounced role in governing both spreading and retraction dynamics.

(2) $$Re=\frac{\rho_{l}v_{0}D_{0}}{\mu_{l}}$$

The Bond number (*Bo*) represents the ratio of gravitational forces to surface tension. When *Bo* ≪ 1, surface tension dominates, and the liquid surface tends to maintain a curved interface, with droplets or bubbles approaching a spherical shape. When *Bo* ≫ 1, gravitational forces dominate, causing the liquid interface to flatten, and droplets may take on a flattened shape.

(3) $$Bo=\frac{\rho_{l}gD_{0}^{2}}{4\sigma_{l}}$$

The Ohnesorge number (*Oh*) is used to describe the relative effects of viscosity, inertia, and surface tension in droplet and interface dynamics. When *Oh* ≪ 1, viscosity is weak, and inertial and surface tension forces dominate the fluid behaviour, making droplets prone to splitting or oscillating. When *Oh* ≫ 1, viscosity is strong, suppressing droplet deformation and splitting, leading to more stable droplet behaviour.

(4) $$Oh=\frac{\mu_{l}}{\sqrt{\rho_{l}v_{0}D_{0}}}=\frac{\sqrt{We}}{Re}$$

The Prandtl number (*Pr*) is used to describe the relative importance of momentum diffusion and thermal diffusion in a fluid. When *Pr* ≫ 1, momentum diffusion is faster than thermal diffusion, indicating that velocity variations within the fluid are more pronounced, but heat transfer occurs more slowly (e.g., in oil-like fluids). When *Pr* ≪ 1, thermal diffusion is faster than momentum diffusion, meaning heat propagates more rapidly than velocity changes (e.g., in liquid metals). When *Pr* ≈ 1, the rates of momentum diffusion and thermal diffusion are similar (e.g., in air and many gases).

(5) $$Pr=\frac{C_{l}\mu_{l}}{k_{s}}$$

According to the study by Teske et al. [25], the evaporation of droplets during the release and impact on the cold substrate is estimated to be less than 0.1%. Therefore, the effect of evaporation is neglected in this study, and the commonly used dimensionless Stefan number is not introduced to describe the experimental conditions. The summary of experimental conditions are listed in Table 1.

To minimize the impact of gravity on the droplet diameter during free fall, the equivalent diameter was used in the experiment. This was achieved by capturing image data of the droplet before impact and treating it as an ellipse according to Equation (6). Where, *D*_0_ represents the equivalent diameter, *D_h_* is the vertical height of the droplet, and *D_w_* is the horizontal width of the droplet. Two porcine bile droplet sizes were investigated in this study, with each size measured and repeated 50 times. In each repetition, a single droplet was allowed to freely fall and then impact the cold substrate. The final equivalent diameters determined were 0.532 mm for the small droplet and 1.028 mm for the large droplet.

(6) $$D_{0}={\left(D_{h}D_{w}^{2}\right)}^{1/3}$$

In this study, the porcine bile droplet impact velocity (*v*_0_) was controlled by adjusting the droplet release height (*H*_0_). The impact velocity was calculated by combining the displacement of the droplet between two consecutive images captured before impact with the time interval between the images. Three release heights were used in the experiment: 10 mm, 20 mm, and 30 mm. The corresponding impact velocities for small droplets were 0.18 m/s, 0.26 m/s, and 0.39 m/s, while for large droplets, the corresponding velocities were 0.25 m/s, 0.38 m/s, and 0.59 m/s. Additionally, the study examined the effect of surface temperature on the impact dynamics and freezing performance. Three different surface temperatures of the cold substrate were used: −10 °C, −15 °C, and −20 °C. The experimental conditions are detailed in Table 2.

## 3. Results and Discussion

### 3.1. Spreading Factor and Rebound Ratio

The spreading factor (*β_moment_*), maximum spreading factor (*β_max_*), and rebound ratio (*β_rebound_*) collectively determine the morphology of a droplet after impact with the cold substrate and the thermal exchange process between the droplet and the substrate surface. The key dynamic characteristics influenced by these factors include the droplet’s spreading speed, maximum spreading range, the efficiency of energy conversion from kinetic energy to heat, as well as changes in the contact area and the distribution of surface temperature gradients. The spreading factor describes the extent of deformation and spreading of the droplet during the impact process. It also determines the droplet’s morphology on the cold substrate and the corresponding impact mode. A low spreading factor may correspond to a rebound or partial adhesion mode, while a high spreading factor may lead to splashing or complete adhesion.

(7) $$\beta_{moment}=\frac{D_{moment}}{D_{0}}$$

where *D_moment_* represents the instantaneous diameter of the droplet obtained from the side view, and *D*_0_ is the equivalent diameter of the droplet.

**Figure 2:**
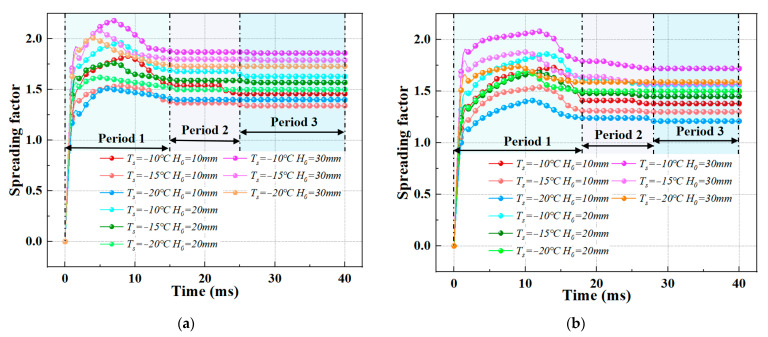
The evolution of the spreading factor (*β_moment_*) for two different porcine bile droplet sizes under varying surface temperatures (*T_s_* = −10 °C, −15 °C, −20 °C) and different release heights (*H*_0_ = 10 mm, 15 mm, 20 mm) (**a**) *D*_0_ = 1.028 mm; (**b**) *D*_0_ = 0.532 mm.

Figure 2 shows the evolution of the spreading factor (*β_moment_*) for two different porcine bile droplet sizes under varying surface temperatures (*T_s_* = −10 °C, −15 °C, −20 °C) and different release heights (*H*_0_ = 10 mm, 15 mm, 20 mm).

General Trend: Despite variations in the spreading factor under different conditions, the evolution curves exhibit similar trends. Specifically, the spreading factor increases sharply at the beginning of the impact process and reaches its maximum value at around 0.005−0.009s after the impact starts. Afterward, the spreading factor gradually decreases. This behaviour aligns with the results observed by Xu et al. [23] and Yang et al. [24] for water droplet impacts.

Three Phases of Spreading Factor Evolution:

- Phase 1 (Rapid Increase): The spreading factor undergoes a significant increase, reaching its maximum.
- Phase 2 (Slow Decrease): The spreading factor gradually decreases after the initial sharp rise.
- Phase 3 (Stabilization): The spreading factor reaches a stable value after the second phase.

Key Observations:

1. Effect of Surface Temperature: As the surface temperature decreases, the time to reach the maximum spreading factor decreases. This could be because a larger temperature difference between the porcine bile droplet and the cold substrate enhances the heat exchange rate, increasing the cooling rate. The faster cooling affects the molecular motion and spreading behaviour of the droplet.
2. Effect of Release Height (Impact Speed): For the same surface temperature, the rate of change of the spreading factor increases with the release height (or impact speed). The increase in impact speed enhances inertial effects (which can be observed through the Reynolds number (*Re*) and Weber number (*We*). A higher impact speed increases the Reynolds number, indicating a stronger inertial effect, and the square relationship of speed significantly increases the Weber number. This means that the porcine bile droplet has a greater ability to overcome surface tension, leading to a greater spread of the droplet.
3. Interaction of Low Release Height and Low Surface Temperature: The combined effect of a lower release height (resulting in lower impact speed) and lower surface temperature reduces the inertial forces and spreading ability of the porcine bile droplet. This also enhances the cooling and freezing effects. This combined effect usually results in a significantly reduced spreading factor. Under extreme low-temperature conditions, the porcine bile droplet may freeze before it reaches its maximum spread, showing a strong inhibition effect.

The maximum spreading factor(*β_max_*) is defined as the ratio of the maximum diameter (or width) that a droplet reaches during its spreading phase after impact on a surface to its initial equivalent diameter before impact. Mathematically, it can be expressed as Equation (8). The influence of the maximum spreading factor on impact dynamics and freezing performance is reflected in various aspects, including the spread of the porcine bile droplet, energy dissipation, heat exchange efficiency, and freezing uniformity. By controlling the initial conditions of the porcine bile droplet (such as velocity and size) as well as the physical properties of the substrate (such as temperature and wettability), the maximum spreading factor can be adjusted. This, in turn, allows for the optimization of freezing performance to meet different engineering requirements.

(8) $$\beta_{max}=\frac{D_{max}}{D_{0}}$$

**Figure 3:**
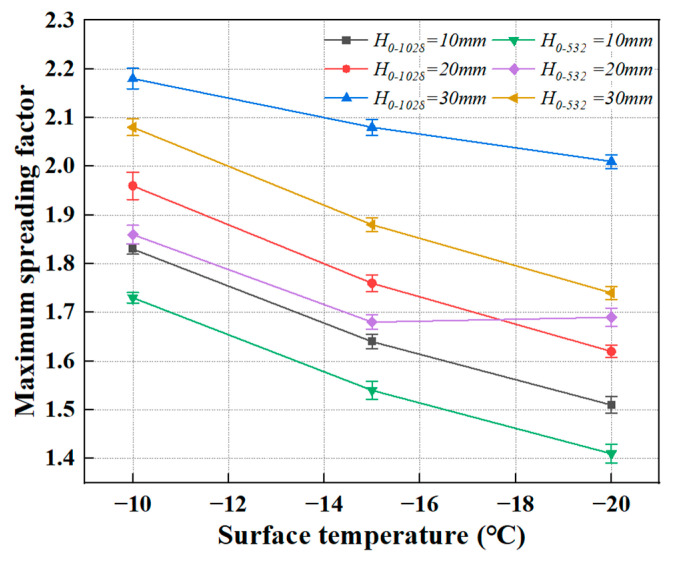
The variation in the maximum spreading factor for two porcine bile droplets of different sizes under different release heights and surface temperatures.

The maximum spreading diameter, *D_max_* is defined as the maximum spread diameter of the porcine bile droplet observed from a side view. Figure 3 illustrates the variation in the maximum spreading factor for two porcine bile droplets of different sizes under different release heights and surface temperatures. It is evident that as the surface temperature of the cold substrate decreases, the maximum spreading factor decreases. Conversely, as the release height (i.e., impact velocity) increases, the maximum spreading factor increases. This can be explained as follows: Lower surface temperatures enhance the cooling and freezing effects. At lower temperatures, the rate of heat transfer between the droplet’s bottom and the substrate increases, which raises the fluid viscosity and weakens the flow, thus reducing the inertial-dominant effect of the droplet. On the other hand, an increase in release height provides greater inertial drive. The gravitational potential energy is converted into more kinetic energy, increasing the inertia (as reflected by larger Reynolds and Weber numbers), which drives the droplet to spread more vigorously upon impact, thereby increasing the maximum spreading factor.

The combined effects of surface temperature and release height determine the maximum spreading behaviour of the porcine bile droplet. The droplet’s dynamics are controlled by a balance between inertial-dominant and cooling-freezing-dominant effects. Under the experimental conditions, the maximum spreading factor ranges from 1.41 to 2.18.

The purple curve shows a different trend from other curves, and intersects with large droplets (red curve, *D*_0_ = 1.028 mm, release height of 20 mm). This cross behaviour reflects that the spreading behaviour of small droplets with medium impact velocity at different temperatures has non-monotonic variation characteristics. Specifically, the mass and kinetic energy of small droplets are relatively small, but the surface area/volume ratio is large, which makes them more sensitive to the change of cold substrate temperature. At higher temperatures, the kinetic energy is slightly insufficient, which limits the spreading; however, at −15 °C to −20 °C, due to the freezing has not yet occurred quickly, the small droplets may have obtained relatively optimal spreading conditions at this stage, resulting in a higher maximum spreading factor than expected. This local ‘enhanced’ spreading behaviour causes the ‘uplift’ of the curve, which eventually intersects with the inertia-dominated large droplet spreading trend.

**Figure 4:**
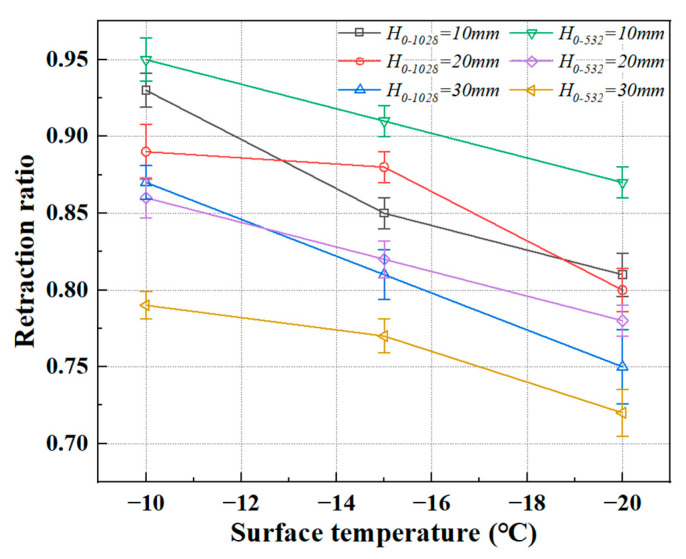
The trend of retraction ratio for two porcine bile droplet sizes during non-instantaneous freezing as a function of surface temperature.

The retraction ratio is defined as the ratio of the droplet’s final equilibrium diameter to its maximum expansion diameter. It reflects the degree of retraction of the droplet after impact, influenced by the combined effects of surface tension and the adhesive force from the cold surface. This parameter has a significant impact on the energy dissipation in the impact dynamics, the transition of impact modes, and the freezing performance, including the freezing initiation time, freezing delay time, and total freezing time. The expression for the recoil rate is given by Equation (9):

(9) $$\beta_{rebound}=\frac{D_{final}}{D_{max}}$$

where, *D_final_* represents the final contact diameter of the droplet, obtained from the side view. The influence of release height and surface temperature on the retraction ratio is shown in Figure 4.

According to the study by Yang et al. [24], freezing can be classified into non-instantaneous freezing and instantaneous freezing. In the preliminary experiments of this study, the freezing processes at *T_s_* = −10 °C, −15 °C and −20 °C were identified as non-instantaneous freezing. When *T_s_* < −21.5 °C, the retraction ratio always remained 1, regardless of the release height. This phenomenon primarily occurs because, at the moment of contact between the porcine bile droplet and the cold substrate, the kinetic energy is rapidly dissipated into heat by the low temperature of the substrate. This heat is used for cooling and freezing, forming a solid “anchoring layer” that prevents further recoil of the porcine bile droplet after spreading, as the frozen layer restricts the droplet’s retraction.

In the three non-instantaneous freezing experiments conducted in this study, for porcine bile droplets of the same size, lower release heights and higher substrate surface temperatures led to larger retraction ratios. For porcine bile droplets of different sizes, at the same release height and cold substrate surface temperature, the retraction ratio of the smaller droplet was generally higher than that of the larger droplet under the same conditions. This is because larger droplets, compared to smaller ones, have greater mass and kinetic energy, resulting in stronger inertial forces that cause the droplet to spread more extensively. However, the recoil process is slower or incomplete due to the inertial effects during spreading. Additionally, smaller droplets have lower volumes and heat capacities. When they contact the cold substrate, the heat within the droplet is absorbed by the substrate more quickly, leading to a faster freezing rate that inhibits further spreading, although the limitation on the recoil process is relatively smaller, leading to a higher retraction ratio.

Figure 4 illustrates the trend of retraction ratios for two porcine bile droplet sizes during non-instantaneous freezing as a function of surface temperature. However, predicting the retraction ratio for given release heights and cold substrate surface temperatures requires further investigation. From the figure, it can be observed that under the conditions of this study, both small and large porcine bile droplets achieve the maximum retraction ratio at *T_s_* = −10 °C and *H*_0_ = 10 mm, with maximum retraction ratios of 0.95 and 0.93, respectively. The minimum retraction ratio is observed at *T_s_* = −20 °C and *H*_0_ = 30 mm, with maximum retraction ratios of 0.75 and 0.72, respectively.

The red curve and the purple curve display crossing trends with the other four curves that follow a more monotonic decrease in retraction ratio with decreasing surface temperature. This crossover behaviour likely results from a nonlinear interplay between inertia and freezing rate under intermediate impact velocities. At higher substrate temperatures (e.g., −15 °C to −10 °C), the inertia of large droplets allows more extensive spreading followed by a noticeable recoil phase, resulting in higher retraction ratios. However, as the surface temperature decreases further (e.g., below −15 °C), rapid solidification suppresses recoil more strongly in large droplets due to their higher thermal mass and slower freezing front propagation.In contrast, the smaller droplets with the same release height experience faster heat loss and solidify more quickly at lower temperatures, but retain higher recoil ability in the mid-temperature range, where freezing is delayed just enough to permit partial retraction. As a result, the retraction ratios of the smaller droplets temporarily exceed those of the larger ones, leading to the observed curve crossings. The phenomenon suggests that a critical balance exists between droplet size, thermal inertia, and freezing kinetics, which can reverse the typical size-dependent retraction behaviour under certain conditions.

### 3.2. Dynamics and Freezing Morphology of Porcine Bile Droplets Characterized by Five Dimensionless Numbers

**Figure 5:**
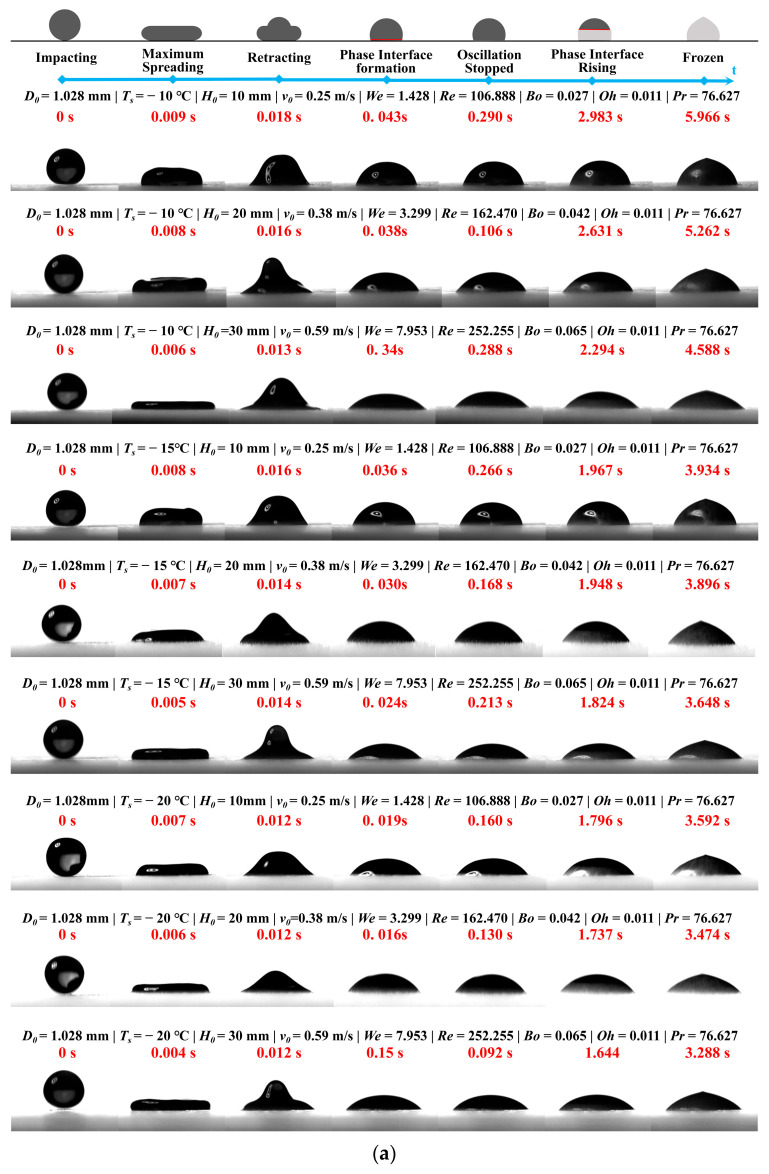

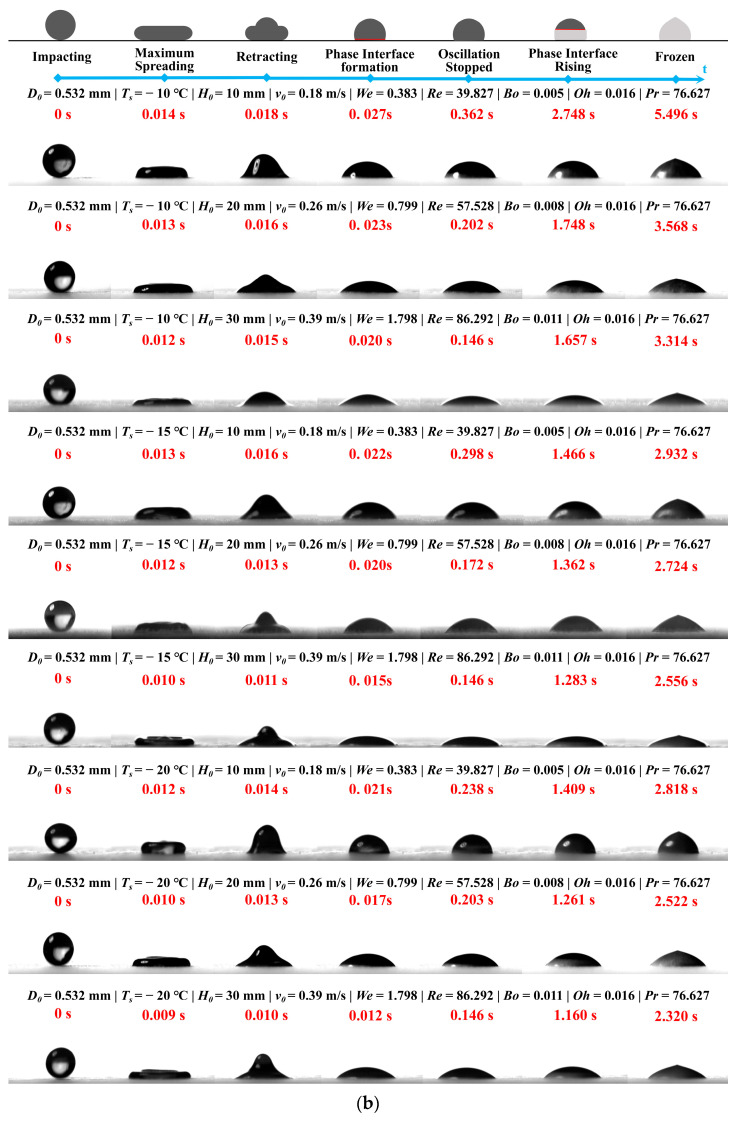
The freezing process of porcine bile droplets impacting a cold substrate under the framework of five dimensionless numbers.(**a**) *D*_0_ = 1.028 mm; (**b**) *D*_0_ = 0.532 mm.

The freezing process of porcine bile droplets impacting a cold substrate under the framework of five dimensionless numbers is illustrated in Figure 5. While viscosity was measured at 20 °C, its minor temperature dependence in the Newtonian regime suggests this simplification does not alter our conclusions on impact dynamics. Two porcine bile droplet sizes (initial diameters *D*_0_ = 1.028 mm and 0.532 mm) were investigated under varying surface temperatures (*T_s_* = −10 °C, −15 °C, and −20 °C) and release heights (*H*_0_ = 10 mm, 20 mm, and 30 mm), corresponding to impact velocities ranging from approximately 0.18 to 0.59 m/s. The influence of five key dimensionless numbers—(*We*, *Re*, *Bo*, *Oh*, *Pr*)—on the impact-to-freezing dynamics and resulting ice crown morphology was systematically examined. The analysis focused on three critical aspects: the evolution of impact dynamics, the nucleation and propagation of the phase boundary, and the final solidified morphology. The regulatory roles of these dimensionless parameters were summarized to elucidate their contributions to the multiphase freezing behaviour.

#### 3.2.1. Impact Dynamics and the Role of Dimensionless Numbers

(1) Maximum Spreading Time and Weber Number (*We*)

For both porcine bile droplet sizes, an increase in the Weber number (*We*, from ~0.38 to 7.95) led to a noticeable reduction in the maximum spreading time (*t_max_*). Specifically, for droplets with *D*_0_ = 1.028 mm, *t_max_* decreased from 0.009 s to 0.006 s, while for *D*_0_ = 0.532 mm, it decreased from 0.014 s to 0.012 s. At the same time, the maximum spreading diameter increased. This behaviour is attributed to the higher impact kinetic energy, which allows the liquid film to overcome surface tension more rapidly and thoroughly, thus shortening the spreading phase and forming a thinner film. Such conditions favor subsequent phase change processes.

(2) Retraction Oscillation Time and Reynolds (*Re*) and Ohnesorge (*Oh*) Numbers

The oscillation period and amplitude during the retraction phase are jointly influenced by the Reynolds number (*Re*) and the Ohnesorge number (*Oh*). Higher *Re* values (e.g., *Re* ≈ 252) correspond to stronger inertial forces, resulting in more intense oscillations. However, the oscillation cessation time (*t_osc_*) is only slightly affected. For instance, in the case of *D*_0_ = 1.028 mm and *We* ≈ 7.95, *t_osc_* ≈ 0.288 s, compared to *t_osc_* ≈ 0.290 s at *We* ≈ 1.43—showing minimal difference.

The oscillation amplitude and energy dissipation are primarily governed by viscous damping, characterized by *Oh* ≈ 0.01. When the porcine bile droplet size is halved (*D*_0_ = 0.532 mm), *Oh* increases slightly to ~0.016, enhancing viscous dissipation. This results in a shorter *t_osc_* under high *We* conditions (e.g., ~0.146 s), confirming that larger *Oh* values facilitate faster attenuation of oscillations.

**Figure 6:**
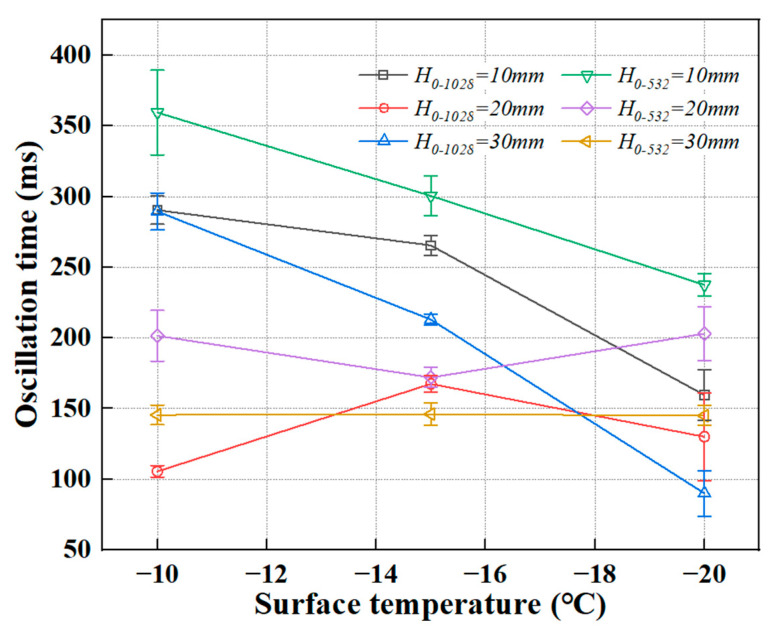
The oscillation time of porcine bile droplets with two different diameters under varying release heights and substrate temperatures.

Figure 6 presents the oscillation time of porcine bile droplets with two different diameters under varying release heights and substrate temperatures. In general, at a fixed substrate temperature, oscillation time tends to decrease as release height increases. Under non-instantaneous freezing conditions, changes in oscillation time with decreasing surface temperature are usually minimal. However, several deviations from typical water droplet behaviour were observed in this study:

First, for smaller porcine bile droplets released from a height of 20 mm, the oscillation time initially decreases and then increases with decreasing substrate temperature. This non-monotonic trend may be attributed to a thermal–dynamic balance occurring near −15 °C. At this temperature, the bottom of the droplet freezes rapidly while the upper portion retains some fluidity. This condition allows efficient energy dissipation through both heat transfer and viscous damping, resulting in faster attenuation of oscillations.

Second, for larger porcine bile droplets released from the same height (20 mm), the oscillation time shows the opposite trend—initially increasing and then decreasing with falling substrate temperature. This can be explained by a competition between freezing and fluid flow. Near −15 °C, the freezing rate and residual fluidity appear to reach a critical balance. Although the porcine bile droplet bottom solidifies quickly, the upper part continues oscillating, allowing the kinetic energy to persist for a longer duration before freezing dominates, which leads to a peak in oscillation time.

Additionally, under the same substrate temperature, porcine bile droplets released from different heights (e.g., 10 mm vs. 30 mm) exhibit negligible variation in oscillation time. This suggests that the initial kinetic energy may lie near a critical threshold, leading to nonlinear behaviour upon impact. Around the 20 mm release height, small perturbations may trigger transitions in freezing dynamics or energy dissipation mechanisms, causing the oscillation time to change more dramatically.

In most cases, the effects of substrate temperature and release height on oscillation time are closely related to the final contact diameter of the porcine bile droplet. Larger final diameters correspond to stronger suppression of oscillation due to surface tension effects.

#### 3.2.2. Interfacial Nucleation and Propagation: Effects of the Prandtl Number (*Pr*) and Bond Number (*Bo*)

(1) Interfacial Nucleation Time

Under identical Weber numbers, the onset of visible interfacial nucleation (denoted as *t_nuh_*) occurs significantly earlier for smaller porcine bile droplets (*D*_0_ = 0.532 mm) than for larger ones (*D*_0_ = 1.028 mm). For instance, at the highest impact energy (*H*_0_ = 30 mm) and substrate temperature *T_s_* = −10 °C, *t_nuh_* is approximately 0.020 s for the smaller droplet, while the larger droplet exhibits a delayed nucleation at around 0.34 s. This difference is primarily attributed to the higher surface-area-to-volume ratio (S/V) of smaller droplets, which enhances heat diffusion and accelerates the onset of phase transition. The Prandtl number remains constant (*Pr* ≈ 76.6), indicating that thermal diffusivity and momentum diffusivity are of similar magnitudes, and that droplet size is the dominant factor influencing nucleation timing.

(2) Interfacial Propagation and Freezing Rate

The time required for the solid–liquid interface to fully propagate to the top of the porcine bile droplet significantly decreases as the substrate temperature drops. Taking *We* ≈ 7.95 as an example, for droplets with *D*_0_ = 0.532 mm, the propagation time decreases from approximately 3.314 s at *T_s_* = −10 °C to 2.320 s at −20 °C—an acceleration of about 30%. A similar trend is observed for larger droplets, with a reduction of approximately 30–40% over the same temperature range. The Bond number (*Bo* ≈ 0.005–0.065) remains extremely low throughout the experiments, indicating that gravitational effects on the thin-film freezing dynamics are negligible. Instead, the propagation process is governed predominantly by thermal conduction and the release of latent heat during phase change.

#### 3.2.3. Final Ice Crown Morphology and Total Freezing Time

(1) Total Freezing Duration

Under all experimental conditions, both an increase in the Weber number (*We*) and a decrease in substrate temperature (*T_s_*) significantly reduce the total freezing time (*t_frozen_*). For larger droplets (*D*_0_ = 1.028 mm), the shortest freezing time observed was approximately 3.288 s at *We* ≈ 7.95 and *T_s_* = −20 °C, while the longest was about 5.966 s at *We* ≈ 1.43 and *T_s_* = −10 °C. For smaller droplets (*D*_0_ = 0.532 mm), *t_frozen_* ranged from roughly 2.320 s to 5.496 s. These results demonstrate that increasing impact energy and lowering the substrate temperature act synergistically to accelerate the solidification process, reducing the total freezing time by up to approximately 45%.

**Figure 7:**
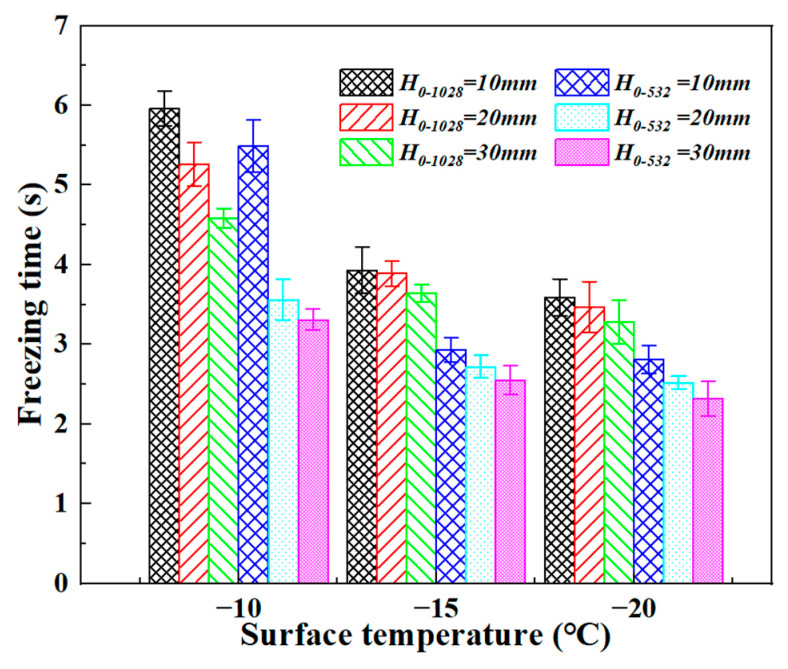
The freezing durations of two porcine bile droplet sizes under various release heights (impact velocities) and substrate temperatures.

The freezing durations of two porcine bile droplet sizes under various release heights (impact velocities) and substrate temperatures are summarized in Figure 7. Under the experimental conditions of this study, all freezing times were below 6 s, indicating a relatively rapid solidification process. Particularly, at a substrate temperature of −20 °C and a release height of 30 mm (corresponding to impact velocities of 0.59 m/s and 0.39 m/s for large and small droplets, respectively), the non-instantaneous freezing times were measured as 3.276 s and 2.316 s, respectively.

Several trends are evident regarding the influence of porcine bile droplet size, impact velocity, and substrate temperature on the freezing time. First, larger droplets generally exhibited longer freezing durations. This effect is attributed to the nonlinear relationship between droplet volume and heat transfer: as droplet size increases, both thermal mass (Q = m·C·ΔT, proportional to volume, where, Q: total heat transferred, m: mass of the droplet, C: specific heat capacity of the liquid) and the distance over which heat must be conducted increase, leading to slower internal heat dissipation and prolonged freezing. Although larger droplets also present a greater contact area with the substrate—potentially enhancing conductive heat transfer (Q = k·A·ΔT·t, ΔT: temperature difference between the droplet and the cold substrate, k: thermal conductivity of the liquid, A: effective contact area between the droplet and the substrate, t: heat conduction time)—this increase is typically insufficient to fully offset the added thermal mass.

Second, freezing time decreased with increasing release height under non-instantaneous freezing conditions. Greater release heights imparted higher kinetic energy and impact velocity to the porcine bile droplets, resulting in broader spreading upon impact. This increase in contact area facilitated faster heat transfer to the substrate. Additionally, the formation of thinner liquid films under high-speed impact reduced the internal heat transfer distance, further accelerating solidification.

Third, freezing time consistently decreased as the substrate temperature was lowered. A larger temperature difference between the porcine bile droplet and substrate (ΔT) enhanced the driving force for heat transfer, enabling faster extraction of thermal energy from the droplet and accelerating the phase transition.

Lastly, the effect of low substrate temperature on freezing time was more pronounced for smaller porcine bile droplets. In contrast, the freezing times of larger porcine bile droplets were less sensitive to substrate cooling. At higher impact velocities, lower substrate temperatures more effectively suppressed droplet recoil by promoting rapid solidification, thereby expediting the freezing process.

(2) Macroscopic Ice Crown Morphology

At low Weber numbers (*We* < 1), the resulting ice structures resembled nearly spherical caps with smooth surfaces and minimal recoil-induced texturing. In contrast, at higher Weber numbers (*We* > 3), thinner and more flattened ice layers were formed, featuring prominent recoil patterns and sharp edge peaks. Smaller porcine bile droplets tended to form more uniform and regular thin-film ice crowns, whereas larger porcine bile droplets, due to their higher thermal capacity, often exhibited peripheral ripples and slight morphological irregularities.

Throughout the porcine bile droplet impact and freezing process on cold substrates, the interplay of five dimensionless numbers governed the dynamics and phase transition behaviours. (i) The Weber number (*We*) primarily dictated the initial spreading behaviour and film thickness, thereby directly influencing the rate of conductive heat transfer. (ii) The Reynolds number (*Re*) and Ohnesorge number (*Oh*) jointly controlled the energy dissipation during recoiling and the attenuation of oscillations. (iii) The Prandtl number (*Pr*) remained approximately constant, ensuring consistent thermal diffusivity across conditions. (iv) The Bond number (*Bo*) was negligible, indicating minimal gravitational influence on the freezing morphology. Finally, porcine bile droplet diameter (*D*_0_) played a critical role by altering the surface-area-to-volume ratio and thermal mass, which significantly affected the timescales of interfacial nucleation and propagation.

This framework offers a quantitative perspective for understanding the rapid freezing behaviour of biological droplets on supercooled substrates.

## 4. Conclusions

This study experimentally investigated the impact dynamics and freezing behaviour of porcine bile droplets on cold substrates. The main findings are summarized as follows:

(1) Impact Dynamics:

The maximum spreading factor of porcine bile droplets was jointly influenced by inertial forces (Weber and Reynolds numbers) and substrate-induced cooling. Higher impact velocities (*We* > 3) significantly enhanced spreading, while lower substrate temperatures suppressed recoiling by accelerating thermal dissipation. The porcine bile droplet retraction ratio increased with decreasing porcine bile droplet size and increasing substrate temperature, reaching a maximum of 0.95 for small droplets (*D*_0_ = 0.532 mm).

(2) Freezing behaviour:

The total freezing time was strongly dependent on porcine bile droplet size, substrate temperature, and impact velocity. Larger droplets (*D*_0_ = 1.028 mm), with greater thermal capacity, exhibited freezing durations 30–40% longer than smaller ones. The shortest freezing times—2.32 s for small droplets and 3.28 s for large ones—were achieved at a substrate temperature of −20 °C, demonstrating the synergistic effect of low temperature and high impact speed in accelerating solidification.

(3) Dimensionless Number Effects:

The Weber number primarily governed initial spreading and film thickness. The Reynolds and Ohnesorge numbers regulated the damping of recoiling oscillations. The Prandtl number remained nearly constant, ensuring consistent thermal diffusivity. The Bond number was negligible, indicating that gravitational effects were minimal. Porcine bile droplet size, through variations in surface area-to-volume ratio and thermal mass, significantly influenced the rate of interfacial nucleation and freezing front propagation.

(4) Engineering Relevance:

These findings provide critical insights for optimizing low-temperature processing techniques of porcine bile, such as freeze-drying and spray-freeze-drying. Additionally, the results offer a novel perspective for multiphase heat transfer studies involving complex biological fluids.

## Figures and Tables

**Table 1:** Summary of the physical properties of porcine bile. Annotation: all tests are at 20 °C, rheological tests confirmed Newtonian behaviour within the shear rate range of 100–1000 s^−1^ (coefficient of variation < 3%), justifying the use of constant viscosity in calculations.

Density	Dynamic Viscosity	Surface Tension	Specific Heat Capacity
993.6 kg/m^3^	2.389 mPa·s	0.04471 N/m	19870 J/(kg·K)

**Table 2:** Summary of experimental conditions.

*D*_0_	*T_s_*	*H*_0_	*v*_0_	*We*	*Re*	*Bo*	*Oh*	*Pr*
1.028 mm	−10 °C	10 mm	0.25 m/s	1.428	106.888	0.027	0.011	76.627
		20 mm	0.38 m/s	3.299	162.470	0.042		
		30 mm	0.59 m/s	7.953	252.255	0.065		
	−15 °C	10 mm	0.25 m/s	1.428	106.888	0.027		
		20 mm	0.38 m/s	3.299	162.470	0.042		
		30 mm	0.59 m/s	7.953	252.255	0.065		
	−20 °C	10 mm	0.25 m/s	1.428	106.888	0.027		
		20 mm	0.38 m/s	3.299	162.470	0.042		
		30 mm	0.59 m/s	7.953	252.255	0.065		
0.532 mm	−10 °C	10 mm	0.18 m/s	0.383	39.827	0.005	0.016	
		20 mm	0.26 m/s	0.799	57.528	0.008		
		30 mm	0.39 m/s	1.798	86.292	0.011		
	−15 °C	10 mm	0.18 m/s	0.383	39.827	0.005		
		20 mm	0.26 m/s	0.799	57.528	0.008		
		30 mm	0.39 m/s	1.798	86.292	0.011		
	−20 °C	10 mm	0.18 m/s	0.383	39.827	0.005		
		20 mm	0.26 m/s	0.799	57.528	0.008		
		30 mm	0.39 m/s	1.798	86.292	0.011		

## Data Availability

Data are contained within the article.
